# Lnc-DARVR/miR-365-1-5p/LAMB1 axis regulates rotavirus replication via the complement C3 pathway

**DOI:** 10.1128/jvi.02114-24

**Published:** 2025-04-16

**Authors:** Xiaopeng Song, Lida Yao, Yan Li, Jinlan Wang, Chenxing Lu, Jinmei Li, Qingmei Leng, Xianqiong Tang, Xiaoqing Hu, Jinyuan Wu, Rong Chen, Xiaochen Lin, Jun Ye, Xiangjun Kuang, Guangming Zhang, Maosheng Sun, Yan Zhou, Hongjun Li

**Affiliations:** 1 Institute of Medical Biology, Peking Union Medical College Institute of Medical Biology, Yunnan Key Laboratory of Vaccine Research and Development on Severe Infectious Disease, Chinese Academy of Medical Sciences 165063, Kunming, China; University of Kentucky College of Medicine, Lexington, Kentucky, USA

**Keywords:** lncRNA, miRNA, LAMB1, ceRNA, infection, complement system

## Abstract

Antiviral effectors and cytokines are critical components of host innate immunity. However, the regulatory mechanisms governing the roles of these molecules in host–virus interactions are still unclear. Although long non-coding RNAs (lncRNAs) have been recognized as key players in various biological processes, their involvement in the complement system of host antiviral defenses remains to be explored. In this study, we discovered a novel, unannotated lncRNA, called DARVR. DARVR was found to be an intergenic lncRNA and inhibited rotavirus (RV) replication in MA104 cells. Mechanistically, we found that complement 3 (C3) was upregulated following RV infection in a LAMB1-dependent manner. However, LAMB1 expression was downregulated by miR-365-1-5p, resulting in the inhibition of the C3-mediated antiviral reaction. However, DARVR functioned as a competing endogenous RNA against miR-365-1-5p, promoting the expression of LAMB1 and thereby enhancing C3 activity and inhibiting RV replication. These results not only provide evidence demonstrating the involvement of lncRNAs in the regulation of RV infection but also highlight the role of complement factors in host innate immunity.

**IMPORTANCE:**

Long non-coding RNAs (lncRNAs) play versatile and critical roles in host–virus interactions, offering significant potential for developing targeted therapies to prevent or treat viral infections. Despite their importance, the involvement of lncRNAs in rotavirus infection remains underexplored. This study identifies a novel lncRNA that enhances complement factor C3 activity through the competing endogenous RNA (ceRNA) mechanism, effectively inhibiting rotavirus replication across different subtypes. These findings underscore the complex molecular interplay regulating complement factor activity during rotavirus infection and provide valuable insights into the host's antiviral mechanisms. This research paves the way for innovative therapeutic strategies targeting lncRNAs and complement factors to combat viral infections more effectively.

## INTRODUCTION

Long non-coding RNAs (lncRNAs) are a class of RNA molecules, typically exceeding 200 nucleotides in length, that cannot be translated into proteins (1). When lncRNAs were first discovered, they were considered genetic noise (2). However, subsequent studies demonstrated the key roles of lncRNAs in a wide range of biological processes (3). In addition to their roles in neurological diseases and cancer, lncRNAs have been found to participate in host–virus interactions. Viruses are known to use host lncRNAs to enhance their proliferation. For example, vesicular stomatitis virus (VSV) influences host metabolism to promote viral infection by hijacking the host lncRNAs ACOD1 and AANCR, which modulate innate antiviral responses (4, 5). Furthermore, lncRNA HULC encourages the release of hepatitis C virus (HCV) particles by supporting the loading of the HCV core protein onto lipid droplets (6). However, host lncRNAs can also protect the host against viral infections. For instance, lnc-zc3h7a promotes RIG-I-mediated antiviral responses following VSV infection (7). Although lnc-TALC does not directly modulate host–virus interactions, it promotes the production of complement 5 (C5) in microglia to enhance antiviral effects (8). Interestingly, some studies have highlighted the role of certain lncRNAs in rotavirus (RV) infection. Specifically, one study showed that lncRNA SLC7A11-AS1 facilitates RV infection by targeting the cystine/glutamate antiporter SLC7A11 and inducing ferroptosis (9). However, the interactions between the complement system and lncRNAs during RV infection remain unclear.

The complement cascade, also known as the complement system, is a vital component of the innate immune system. It complements the action of antibodies and targets the cell membranes of pathogens, enhancing their clearance and promoting inflammation (10). Remarkably, the complement system remains unaltered throughout an individual’s lifetime. The complement system can be activated by three primary biochemical pathways: the classical complement pathway, the alternative complement pathway, and the lectin pathway (11). Regardless of the trigger, the central event in the complement pathway is the conversion of C3 to C3b and C3a, which marks a crucial step in pathogen elimination (12). However, given the central role of C3 in immune surveillance, C3 is often the target of the immune evasion strategies employed by pathogens. For example, the NSP1 protein of porcine epidemic diarrhea virus (PEDV) and HCV inhibits C3 complement production to help the viruses escape replication constraints (13, 14). C3 is classified as a type I acute-phase protein, and it is upregulated by a host of inflammatory factors (15). However, reports on the regulation of C3 by host molecules following viral infection are fairly limited.

LAMB1 is a member of the laminin family, and it serves as the primary non-collagenous constituent of basement membranes. However, LAMB1 has been implicated in a diverse array of biological processes, including cell adhesion, neurite outgrowth, and metastasis (16). Interestingly, studies have also demonstrated that LAMB1 participates in inflammatory and complement cascades (17, 18). Some studies have also reported the interaction between LAMB1 and cytokines such as interleukin-1 (IL-1), IL-6, and tumor necrosis factor-alpha (TNF-α), which upregulate C3 (19). In fact, IL-1β and IL-6 synergistically enhance C3 mRNA levels and protein secretion in rat hepatoma cells (20). However, credible studies proving the direct relationship between LAMB1 and C3 are currently lacking.

Recent studies suggest that the interactions between miRNAs and mRNAs are not unidirectional. Instead, various types of RNA molecules—including pseudogenes, lncRNAs, and circular RNAs—compete for miRNA binding, thereby modulating mRNA activity (21). These competitive endogenous RNAs (ceRNAs) act as molecular sponges for miRNAs via their miRNA-binding sites (22). For example, lncRNA AANCR was found to promote MITA expression during *Siniperca chuatsi* rhabdovirus (SCRV) infection by competitively binding to miR-200, thus establishing a ceRNA network, and lncRNA CYLD reversely inhibits type-I interferon through ceRNA mechanism in bovine viral diarrhea virus (BVDV) infection (4, 23). Transcriptome sequencing of RV-infected and RV-uninfected MA104 cells led to the discovery of a novel, unannotated lncRNA: TCONS_00006666. Our research showed that it is a down-regulated anti-rotavirus long non-coding RNA*,* thus we named it DARVR. Further research on the mechanism by which DARVR regulates RV infection revealed the presence of a ceRNA loop between DARVR and miR-365-1-5p. Specifically, the findings showed that C3 and LAMB1 co-regulate antiviral responses following RV infection. Although LAMB1 is negatively regulated by miR-365-1-5p, promoting RV replication, lncRNA DARVR can act as a ceRNA and bind to miR-365-1-5p, thus playing a protective role by maintaining C3 expression and activity following RV infection.

## MATERIALS AND METHODS

### Cells and viruses

MA104 cells and Vero cells were cultured in Dulbecco’s modified Eagle medium (DMEM, Gibco, USA) supplemented with 8% fetal bovine serum (FBS; Gibco, USA) at 37°C in 5% CO2. In the stimulation experiments, MA104 cells were challenged with RV at the relevant multiplicity of infection (MOI) values and indicated time points.

The wild ZTR-68 rotavirus strain (G1) was isolated from the feces of a child with diarrhea in Yunnan province (China). The G3 is Simian rotavirus strain SA11, the G4 is porcine rotavirus strain Gottfried, and the G9 is human rotavirus strain Wa. All of them are kept in the Laboratory of Molecular Biology, Chinese Academy of Medical Sciences.

### Plasmid construction

The DARVR sequence of MA104 cells was synthesized by Jin Kairu. The synthesized fragments contain restriction sites BsaBI and BstBI. Then, they were cloned into pcDNA 3.1 (Jin Kairui, Wuhan), while the vector control does not contain the DARVR sequence. The orientations and the sequences of the inserts were verified by restriction digestion and sequencing. The plasmids were extracted through the Fast Pure EndoFree Plasmid Maxi Kit (MD103, Novozant, Nanjing, China).

The sequences of LAMB1 3′ UTR containing the wild-type (WT) or mutant (Mut) binding site of mml-miR-365-1-5p were devised and synthesized by GenePharma (Shanghai, China). The LAMB1 3′ UTR and mutant LAMB1 3′ UTR were, respectively, cloned into pGl3-basic vectors (Jin Kairui, Wuhan).

### RNA-seq

MA104 cells in T25 cell culture flasks were grown to 90% confluence and infected or not infected with ZTR-68 at 0.1 MOI for 24 hours. The cell surface was then washed twice with PBS, 1 mL Trizol was added to each T25, and RNA-seq was performed by oebiotech.

### RNA FISH and immunofluorescence assays

For RNA FISH, miR-365-1-5p was labeled with CY3, and DARVR was labeled with Alexa Fluor 488 (Servicebio, GPD1070). Cells were fixed in 4% paraformaldehyde for 20 min, and then the DARVR probe was added and allowed to hybridize overnight at 37°C. After washing with the saline sodium citrate (SSC) washing buffer, the miR-365 probe was added and allowed to hybridize overnight at 37°C. Then, the cell nuclei were stained with DAPI, and cells were observed under a fluorescence-inverted microscope (Nikon, TE2000-U).

For the immunofluorescence assay, the cells were fixed in 4% paraformaldehyde containing 0.2% Triton-X 100 for 30 min. Then, a purified goat anti-RV antibody was employed as the primary antibody (Institute of Medical Biology, Chinese Academy of Medical Sciences, RVAB2020101). Subsequently, a FITC-conjugated rabbit anti-goat antibody (Cat. No. 305-095-003, Jackson Immune Research, USA) was used as the secondary antibody, and the cells were stained with DAPI before observation, and cells were observed under a fluorescence inverted microscope (Nikon, TE2000-U).

### RNA extraction and RT-PCR

Total cellular RNA was extracted using the TRizol reagent (Invitrogen, 15596018CN). Viral RNA was extracted by MiniBEST Viral RNA/DNA Extraction Kit (Takara, Cat. No. 9766). Then, the HiScript II One Step qRT-PCR SYBR Green Kit (Q222, Novozant, Nanjing, China) was used to detect viral genomic dsRNA. DARVR was detected in MA104 cells and tissues from suckling mice using the HiScript II One Step qRT-PCR SYBR Green Kit (Q221, Novozant, Nanjing, China). The relative expression levels of the target gene were normalized to the expression of β-actin as the internal reference gene. All these primer sequences are in Tables 1 to 3. Relative gene expression data were acquired using the 2^−∆∆Ct^ method.

**TABLE 1:** RT-PCR primers for host

	Forward sequence	Reverse sequence
DARVR	TGCAGTTGGAACTTAGAGTGG	GGACAAAGTTCTGCTGAGAC
Beta-actin	CAGATGTGGATCAGCAAGCAGGAG	CAGTAACAGTCCGCCTAGAAGCAC
MALAT1	GACGCCAGAGCCCTGAATTA	CCCTGCGTCATGGAGTTCAA
U6	CTCGCTTCGGCAGCACA	AACGCTTCACGAATTTGCGT

**TABLE 2:** RT-PCR primers for ZTR-68 NSP3

	Sequence
Forward primer	ACCATCTACACATGACCCTC
Reverse primer	GGTCACATAACGCCCC
Probe	ATGAGCACAATAGTTAAAAGCTAACACTGTCAA

**TABLE 3:** RT-PCR primers for miR-365-1-5p

	Sequence
miR-365 RT	CTCAACTGGTGTCGTGGAGTCGGCAATTCAGTTGAGACATCTG
miR-365 forward primer	ACACTCCAGCTGGGGAGGGACTTTTGGGGGC
miR-365-universal-A reverse primer	TGGTGTCGTGGAGTCG

### RNAi

All siRNAs targeting lncRNA DARVR, LAMB1, and C3 were designed and synthesized by Gene Pharma and were accompanied by corresponding negative controls. The sequences of all these RNA oligonucleotides are shown in Table 4.

**TABLE 4:** siRNAs

	Sense sequence	Antisense sequence
DARVR	GGUCCCUGUUGAACUUAGATT	UCUAAGUUCAACAGGGACCTT
LAMB1-siRNA1	CCCGAGGAUACAGAAUUUATT	UAAAUUCUGUAUCCUCGGGTT
C3	GGUCAACUCACCUGUAAUATT	UAUUACAGGUGAGUUGACCTT

### DNA and RNA transfection

For both DNA (pcDNA 3.1 DARVR) and RNA oligonucleotide transfection, use Lipofectamine 3000 Reagent (Thermo Fisher, L3000008) and refer to the instructions for the use of the product. The miR-365-1-5p mimics sequences are 5′-GAGGGACUUUUGGGGGCAGAUGU-3′. The miR-365-1-5p inhibitor sequences are 5′- ACAUCUGCCCCCAAAAGUCCCUC-3′. The mimics-NC sequences are 5′-UUCUCCGAACGUGUCACGUTT-3′. The inhibitor NC sequences are 5′CAGUACUUUUGUGUAGUACAA3′.

### Dual-luciferase reporter assay

Each plasmid was co-transfected according to the grouping described in Results. Forty-eight hours after transfection, cells were lysed, and a dual luciferase reporter assay system (Promega, E1500) was employed to detect reporter activity. All luciferase activity values were normalized to the Renilla luciferase control. Transfections for each construct in each assay were performed in triplicate. The ratio of Renilla luciferase to firefly luciferase activity was obtained and averaged across the triplicates. LAMB1 3′ UTR is from NCBI 16777 (Gene ID).

### Western blotting

After washing the cells with PBS, the total cellular protein was extracted on ice using RIPA lysis buffer (Beyotime, P0013B). The protein concentration in each sample was then determined using a BCA kit (Beyotime, P0009), and western blot experiments were then performed. Details of antibodies used in the experiments are shown in Table 5.

**TABLE 5:** Antibodies

Antibody	Source	Identifier
LAMB1	Proteintech	Cat No: 23498-1-AP
C3	GeneTex	GTX72994
β-actin	Proteintech	Cat no: 66009-1-Ig
Rabbit IgG	Proteintech	Cat no: 30000-0-AP
RV NSP3	Chinese Academy of Medical Sciences	RVAB2020101
Fluorescein (FITC)-conjugated AffiniPure donkey anti-goat IgG (H + L) (min X Ck,GP,Sy Hms,Hrs,Hu,Ms,Rb,Rat Sr Prot)	Jackson	Code: 705-095-147
HRP-Goat anti-rabbit recombinant secondary antibody (H + L)	Proteintech	Cat no: RGAR001
Multi-rAb HRP-goat anti-mouse recombinant secondary antibody (H + L) Cy3 AffiniPure donkey anti-rabbit IgG (H + L)	Proteintech Jackson	Cat no: RGAM001 Code: 711-165-152

### RNA pull-down

TranscriptAid T7 kit (Thermo Fisher, K0441) was used for *in vitro* transcription of DARVR and antisense-DARVR. Thermo Scientific Pierce RNA 3′ (Thermo Fisher, 20163) was used to label *in vitro* transcripts. RNA Pull Down kit (Gene Create, JKR23004) was used to detect the enrichment of DARVR on miR-365, while the anti-sense DARVR was selected as a positive control. Finally, RT-PCR was used to detect the RNA pull-down experimental products.

### Co-IP

For Co-IP experiments, the protein A/G Magnetic IP/Co-IP Kit (PB201, Novozant, Nanjing, China) was used. The immunoprecipitation antibodies of rabbit IgG and LAMB1 were used to capture the whole protein in rotavirus-infected MA104 cells. Then, the Western blotting experiment was used to detect LAMB1 and C3.

### Animal experiment

The diarrhea model in suckling mice was established using the ZTR-68 strain of rotavirus. Three to four days after the pregnant mice gave birth, the ZTR-68 strain of rotavirus was administered to the suckling mice via gavage. Three days post-infection, the suckling mice exhibited symptoms of diarrhea. Subsequently, the mice were euthanized, and their organs were collected for further analysis.

### Statistical analysis

Data were presented as the mean ± SE of at least three independent experiments. The student’s t-test was used to compare the data between groups. Comparisons among multiple groups were analyzed via one-way analysis of variance (ANOVA), followed by Duncan’s multiple comparison tests. A value of *P* < 0.05 was considered statistically significant.

Pearson correlation test was used to calculate the expression correlation between lncRNA and mRNA based on the differential expression data. Pairs with correlation coefficients not less than 0.8 and *P*-values less than or equal to 0.05 were selected and considered to have a co-expression relationship. The *P*-values were arranged from small to large, and the top 500 were taken to draw the following co-expression network.

For quantification of fluorescence intensity, we use imagej to calculate the average fluorescence intensity of DAPI and FITC in the entire field of view and then calculate the ratio of the average fluorescence intensity of FITC and DAPI.

## RESULTS

### Biological properties of DARVR

**Fig 1:**
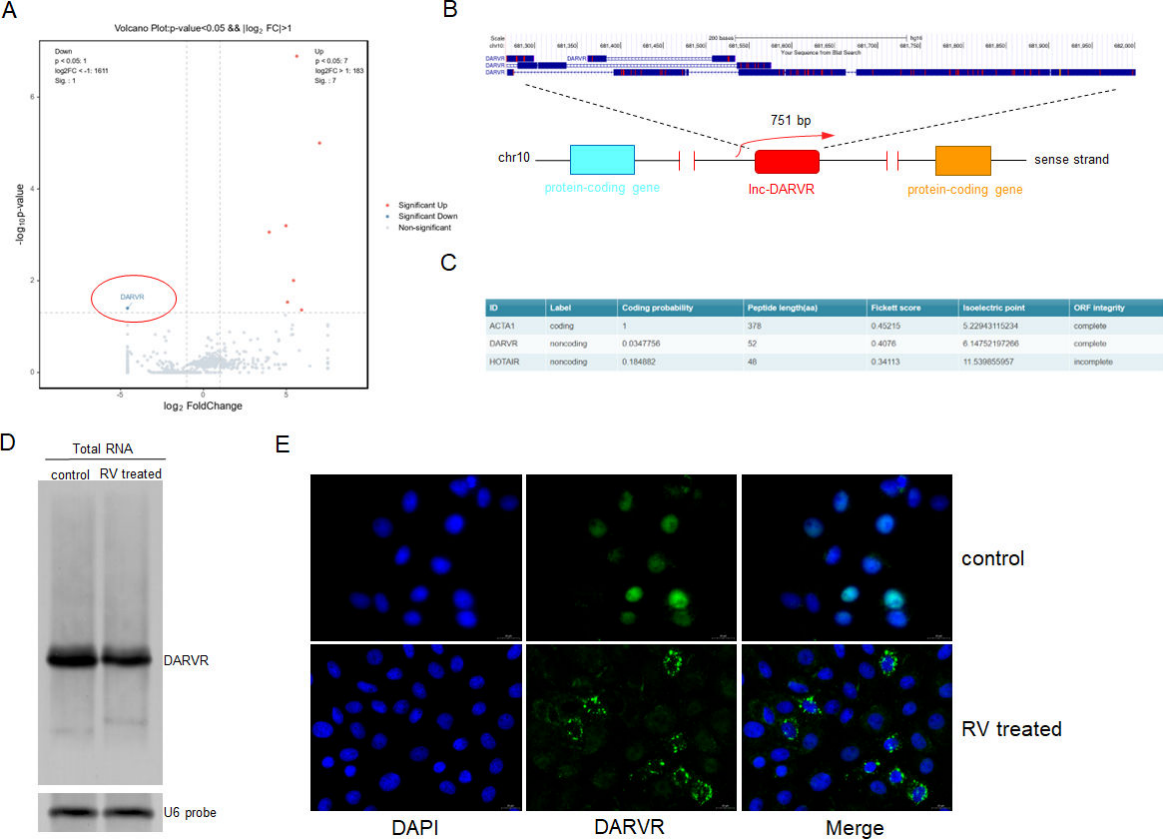
Identification and biological characterization of DARVR. (**A**) RNA-seq of MA104 cells infected with ZTR-68 (MOI = 0.1) for 24 hours. (B) UCSC Genome Browser analysis showed that DARVR is an intergenic lncRNA, 751 base pairs in length, and is located on human chromosome 10. (C) The sequence of DARVR was predicted to be a non-coding RNA after its RNA sequence was analyzed by coding potential calculator 2. (D) Northern blot for +DARVR using the total RNA isolated from uninfected and Rotavirus-infected MA104 cells (ZTR-68, 24 h, MOI = 0.1). U6 RNA served as the loading control. These data are representative of at least three independent experiments. (E) RNA FISH for DARVR in uninfected and RV-infected MA104 cells (ZTR-68, 24 h, MOI = 0.1). These data are representative of at least three independent experiments.

RNA-seq revealed a new lncRNA that was downregulated after rotavirus infection, which we named DARVR (Fig. 1A). To identify the chromosomal location of DARVR, its sequence was uploaded to the UCSC Genome Browser. As shown in Fig. 1A, DARVR is an intergenic lncRNA derived from chromosome 10 (Fig. 1B). Coding potential calculator 2 (CPC2) predicted that DARVR has a low coding potential (Fig. 1C). The presence of DARVR in the total RNA isolated from uninfected and RV-infected MA104 cells (24 h) was confirmed via northern blot, with U6 RNA serving as the loading control (Fig. 1D). In addition, RNA fluorescence *in situ* hybridization (FISH) indicated that partial DARVR migrates from the nucleus to the cytoplasm during RV infection (Fig. 1E).

### DARVR is downregulated during RV infection

**Fig 2:**
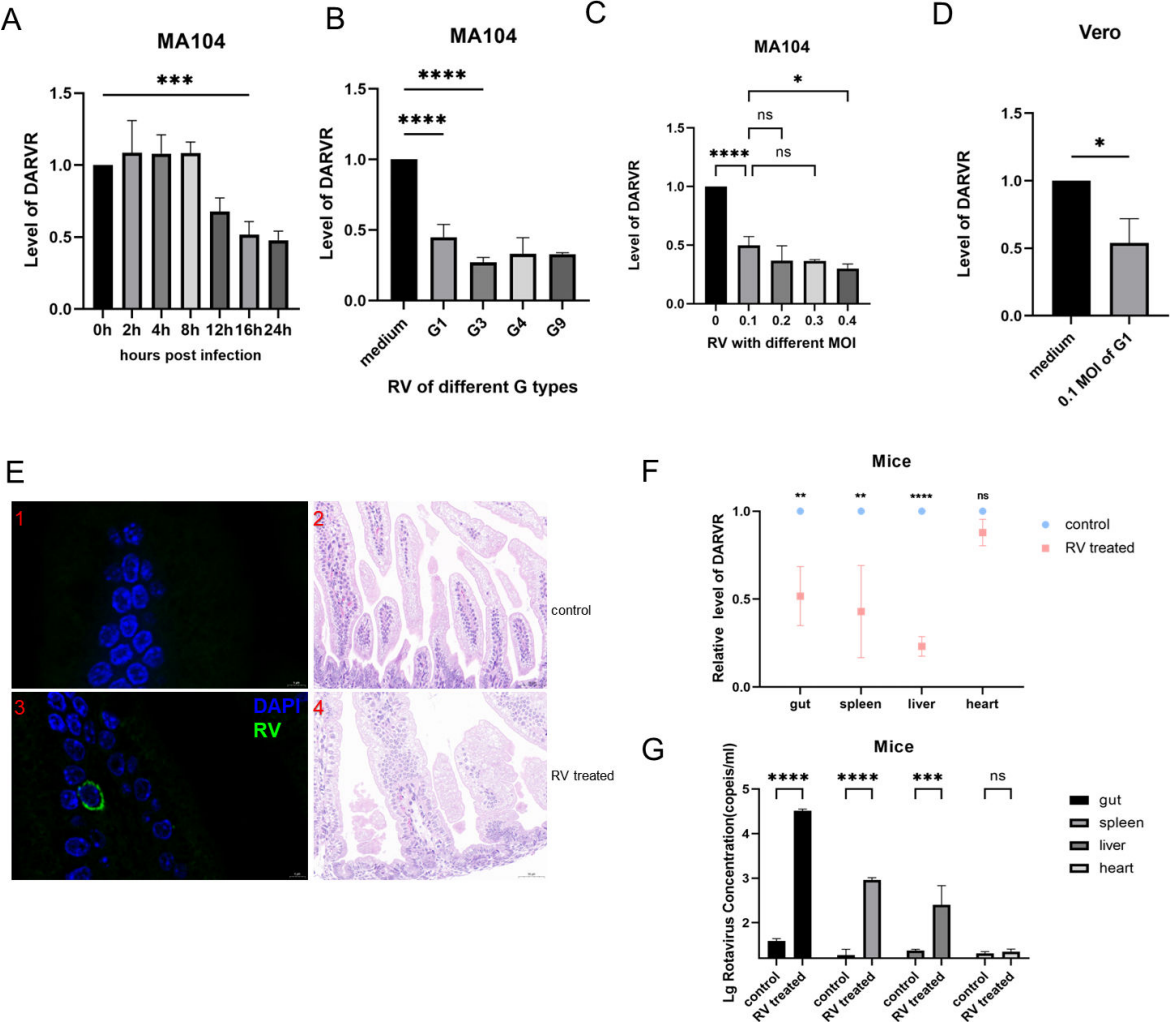
RV infection causes the downregulation of DARVR levels. (**A**) DARVR expression levels at different time points after 0.1 MOI ZTR-68 infection. (B) DARVR expression levels after infection with different strains of RV with 0.1 MOI. (C) DARVR expression levels in MA104 cells after infection with the RV strain ZTR-68 at different MOI values. (D) DARVR expression levels in Vero cells after infection with ZTR-68 of 0.1 MOI values. (E) Immunofluorescence (1,3) and H&E (2,4) staining of intestinal tissue in uninfected and RV-infected 3-day-old suckling mice. (F) Detection of DARVR expression levels in the gut, spleen, liver, and heart of uninfected and RV-infected suckling mice via RT-PCR. (G) Detection of viral copy numbers in the gut, spleen, liver, and heart of suckling mice infected with 0.1 MOI ZTR-68 for 48 hours or not infected. The student’s t test was used for comparison between two groups, and comparisons among multiple groups were analyzed via one-way analysis of variance (ANOVA). Data are presented as the mean ± SD of three independent experiments (ns, no significance difference. *P < 0.05. **P < 0.01. ***P < 0.001. ****P < 0.0001).

To explore the time points at which DARVR is downregulated during RV infection in MA104 cells, total RNA from MA104 cells infected with 0.1 MOI RV for 0–24 h was extracted, and RT-PCR was performed. DARVR was significantly downregulated after 16 h of infection, and this downregulation persisted until 24 h after infection (Fig. 2A). Furthermore, the degree of DARVR downregulation varied depending on the strain of RV with 0.1 MOI (i.e., G1, G3, G4, and G9) (Fig. 2B). To explore the relationship between decreased DARVR expression and RV infection, MA104 cells were infected with RV at different MOI values. Interestingly, DARVR was downregulated more after infection with 0.4 MOI of RV compared to 0.1 MOI of RV. However, there was no statistical difference between 0.2 and 0.3 MOI of RV and 0.1 MOI of RV (Fig. 2C). Moreover, RV will also cause a downward adjustment of Vero DARVR (Fig. 2D).

Furthermore, the potential downregulation of DARVR was examined *in vivo*. Suckling mice (3–4 days old) were infected with RV via oral gavage. Immunofluorescence analysis of intestinal tissue (Fig. 2E1 and E3) demonstrated that RV had infected the intestines of the mice and induced intestinal damage (Fig. 2E2 and E4). Total RNA was extracted from the gut, spleen, liver, and heart of the mice, and DARVR expression was quantified using RT-PCR. The results showed that DARVR was downregulated in the gut, liver, and spleen after RV infection, but its expression in the heart was unaffected (Fig. 2F). Besides, we measured the viral copy number in the gut, spleen, liver, and heart of suckling mice infected with 0.1 MOI of ZTR-68 for 48 hours or left uninfected (Fig. 2G).

### DARVR inhibits RV replication

**Fig 3:**
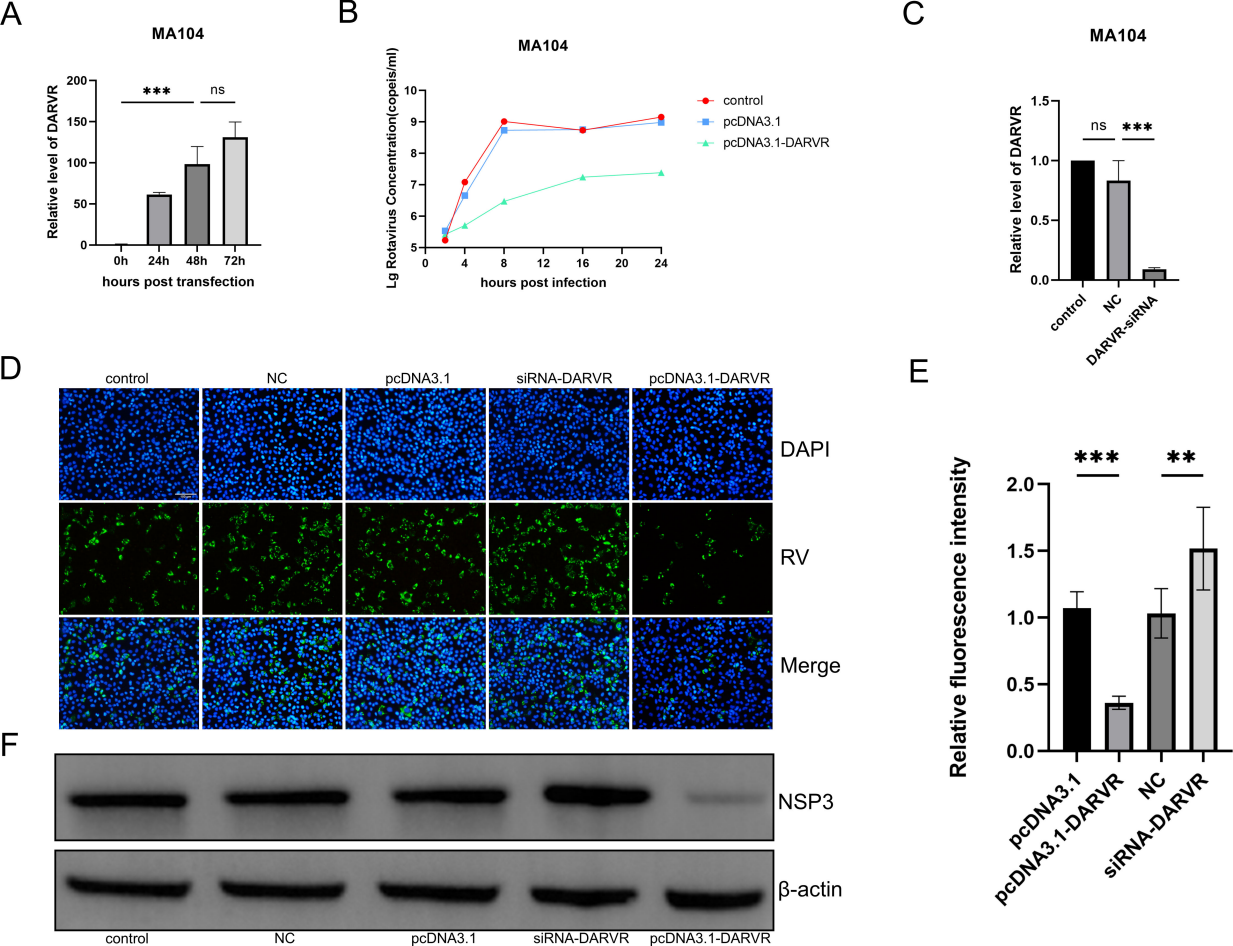
DARVR inhibits RV replication. (**A**), DARVR levels at different time points following pcDNA3.1-DARVR transfection. (B) RV replication kinetics within 24 h after pcDNA3.1-DARVR transfection (ZTR-68, 24 h, MOI = 0.1). (C) siRNA experiments against DARVR. (D) RV replication at 16 h after DARVR inhibition and overexpression in MA104 cells was examined using immunofluorescence assays. (E) The relative fluorescence intensity was calculated by counting infected and uninfected cells using an immunofluorescence assay. (F) RV NSP3 was detected using a western blot after 16 h of infection. Comparisons among multiple groups were analyzed via one-way analysis of variance (ANOVA). Data are presented as the mean ± SD of three independent experiments (ns, no significance difference. ***P < 0.001).

To elucidate the role of DARVR during RV infection, rhesus DARVR was overexpressed in MA104 cells using the eukaryotic expression vector pcDNA3.1. The expression levels of DARVR peaked at 72 h post-transfection. However, there was no significant difference between the expression levels at 48 h and 72 h (Fig. 3A). Thus, 48 h was selected as the time point for RV infection. At 48 h post-transfection, MA104 cells were infected with RV, and total RNA was extracted from these cells to detect RV replication. The amount of virus in the cells was largely comparable between the infected and uninfected groups at the 2 h time post-infection point, which corresponds to the stage of virus entry. However, at 4 h, the amount of virus in the DARVR overexpression group was significantly higher than that in the control group. Within 24 h of infection, the amount of virus in the DARVR overexpression group became consistently lower than that in the control group (Fig. 3B).

To explore whether DARVR inhibition could promote RV replication, DARVR siRNA was designed and transfected into MA104 cells, and these cells were infected with RV at 48 h post-transfection (Fig. 3C). Given that RV replication plateaus around 16–24 h, immunofluorescence experiments were performed on MA104 cells at 16 h post-RV infection. The results showed that RV replication was slightly increased in MA104 cells treated with DARVR siRNA, while the replication was significantly inhibited after DARVR overexpression (Fig. 3D). Subsequently, the relative fluorescence intensity of 3D was calculated (Fig. 3E). The non-structural protein NSP3 is abundantly expressed in RV during its replication stage and can be used to estimate its replication status. Thus, total protein was extracted from MA104 cells under the aforementioned treatment conditions, and western blotting was performed. Unsurprisingly, following DARVR siRNA treatment, the amount of NSP3 was found to be slightly higher than that in the NC group. Meanwhile, following pcDNA-3.1 DARVR transfection (i.e., DARVR overexpression), the amount of NSP3 was significantly lower than that in the pcDNA3.1 (control) group (Fig. 3F). These results showed that DARVR inhibits RV replication.

### DARVR sponges off mir-365-1-5p during RV infection

**Fig 4:**
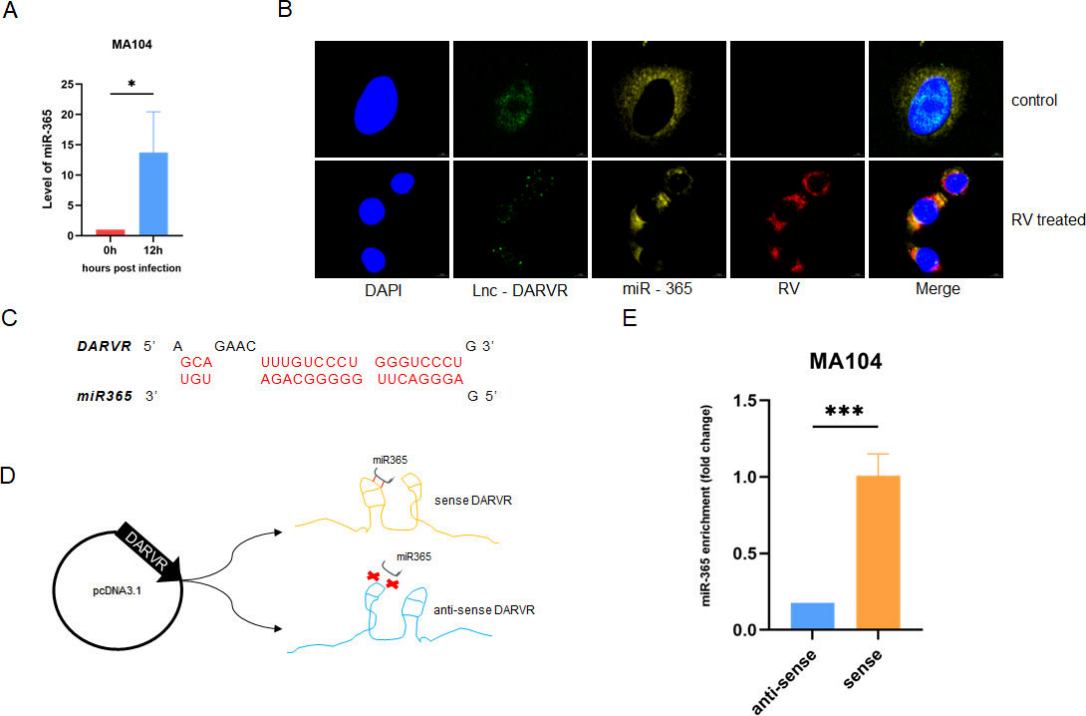
DARVR exits the nucleus and binds to miR-365-1-5p. (**A**), Expression levels of miR-365-1-5p after RV infection (ZTR-68, 24 h, MOI = 0.1). (B) Confocal microscopy showing DARVR and miR-365-1-5p expression at 16 h in infected and uninfected cells (ZTR-68, 24 h, MOI = 0.1). (C) Binding sites between DARVR and miR-365-1-5p. (D) Schematic diagram of the RNA pull-down assay in MA104 cells, in which miR-365-1-5p was enriched using *cis* and *trans* DARVR. (E) RT-PCR assay for miR-365-1-5p was performed after the pull-down assay. The student’s t test was used for comparison between two groups. Data are presented as the mean ± SD of three independent experiments (**P < 0.01. ***P < 0.001).

Research has shown that miRNAs bind to their target RNAs and thereby inhibit the translation of these RNAs (24). In this study, target prediction analysis suggested that DARVR may interact with miR-365-1-5p. Interestingly, a previous study showed that mir-365-1-5p can be upregulated by bovine RV (25). Our findings demonstrated that human RV can also upregulate miR-365-1-5p (Fig. 4A). After examining miR-365-1-5p and DARVR expression during RV infection, we performed FISH assays and used confocal microscopy to explore the direct interaction between these molecules. The findings showed that after RV infection, DARVR exited the nucleus and bound to miR-365-1-5p (Fig. 4B). Furthermore, the tool miRanda predicted the presence of binding sites between miR-365-1-5p and lncRNA DARVR (Fig. 4C). To detect the binding specificity between DARVR and miR-365-1-5p, sense DARVR and anti-sense DARVR were expressed in MA104 cells using the pcDNA3.1 vector. RNA pull-down assays targeting miR-365-1-5p were performed, and sense DARVR was found to have a high enrichment of miR-365-1-5p, while the anti-sense DARVR was barely enriched in miR-365-1-5p (Fig. 4D and E). This confirmed that DARVR can bind to mir-365-1-5p during RV infection, acting as a molecular sponge.

### Interactions between DARVR and miR-365-1-5p modulate RV replication

**Fig 5:**
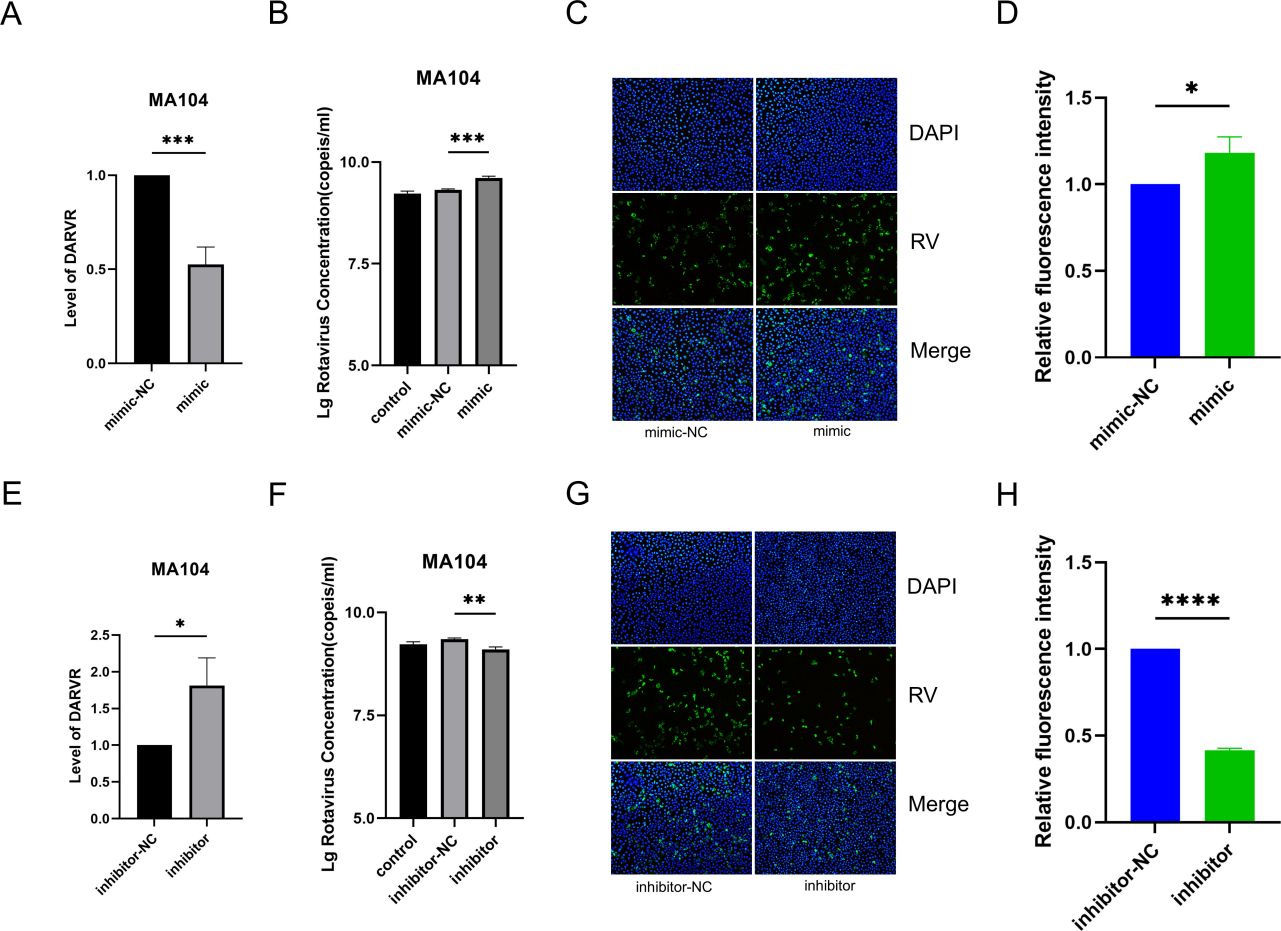
Impact of miR-365-1-5p on DARVR and its role in RV replication. (**A**) Effect of miR-365-1-5p overexpression on DARVR expression. (B) Effect of miR-365-1-5p overexpression on RV (ZTR-68, MOI = 0.1) replication at 16 h post-infection, examined using RT-PCR. (C) Effect of miR-365-1-5p overexpression on RV (ZTR-68, MOI = 0.1) replication at 16 h post-infection, examined using immunofluorescence assays. (D) The relative fluorescence intensity of RV and DAPI in each group was quantified using ImageJ (normalized to the control group). Data are presented as the mean ± SD of three independent experiments. Statistical significance was analyzed using a two-tailed Student’s t-test (*P < 0.05). (E) Effect of miR-365-1-5p inhibition on DARVR expression. (F) Effect of miR-365-1-5 inhibition on RV (ZTR-68, MOI = 0.1) replication at 16 h post-infection, examined using RT-PCR. (G) Effect of miR-365-1-5p inhibition on RV (ZTR-68, MOI = 0.1) replication at 16 h post-infection, examined using immunofluorescence assays. (H) The relative fluorescence intensity of RV and DAPI in each group was quantified using ImageJ (normalized to the control group). The Student’s t test was used for comparison between two groups, and comparisons among multiple groups were analyzed via one-way analysis of variance (ANOVA). Data are presented as the mean ± SD of three independent experiments. (*P < 0.05, **P < 0.01, ***P < 0.001, ****P < 0.0001).

After confirming the binding between miR-365-1-5p and DARVR during RV infection, we examined the effect of this interaction on DARVR expression. The miR-365-1-5p mimics was transfected into MA104 cells, and after 48 h, DARVR was downregulated in these cells (Fig. 5A). Notably, at 48 h following mimic transfection, the cells were infected with RV. After 16 h of infection, immunofluorescence assays were performed, and total cellular RNA was also extracted. Mimic transfection inhibited the expression of DARVR and ultimately promoted RV replication (Fig. 5B). Immunofluorescence experiments showed that the number of RV particles was higher in the mimic group than in the mimic-NC group (Fig. 5C), and quantitative analysis confirmed these findings (Fig. 5D). Subsequently, we tested whether DARVR would be upregulated when miR-365-1-5p is inhibited, resulting in reduced RV replication. To this end, an miR-365-1-5p inhibitor was transfected into MA104 cells under the same treatment conditions. Unsurprisingly, DARVR expression was upregulated (Fig. 5E), and RV replication was inhibited (Fig. 5F through H). Overall, these findings showed that miR-365-1-5p could inhibit the expression of DARVR to promote RV replication.

### miR-365-1-5p inhibits LAMB1 to promote RV replication

**Fig 6:**
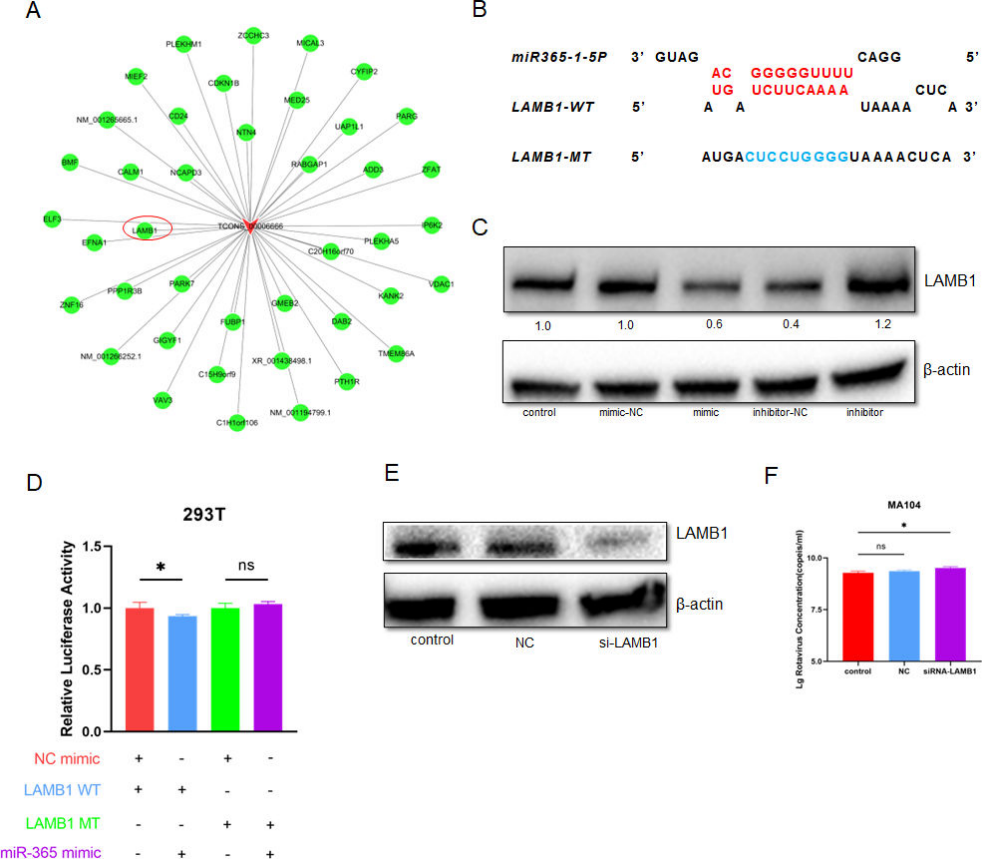
miR-365-1-5p promotes RV replication by suppressing LAMB1. (**A**) RNA-seq reveals that LAMB1 is the closest co-expressed gene to DARVR. (B) Interactions between miR-365-1-5p and the LAMB1 wild-type and mutant 3′ UTRs were detected using a dual luciferase reporter assay in 293T cells. (C) At 72 h after transfection with a miR-365-1-5p mimic and inhibitor, the total cell protein was extracted, and the changes in LAMB1 protein expression were detected via western blotting. (D) The effect of LAMB1 inhibition was detected by western blotting, and three siRNAs targeting LAMB1 were transfected. (E) The effect of LAMB1 inhibition on RV replication at 16 h post-infection was examined using RT-PCR. RV (ZTR68, MOI = 0.1) replication was detected at 16 h post-infection in MA104 transfected with siRNAs and infected with RV at 72 h after transfection. Comparisons among multiple groups were analyzed via one-way analysis of variance (ANOVA). Data are presented as the mean ± SD of three independent experiments. (*P < 0.05).

RNA-seq results show that LAMB1 is a co-expressed gene of DARVR (Fig. 6A). Typically, miRNAs degrade the target mRNA by binding to their 3′ untranslated regions (UTRs). To know whether miR-365-1-5p will lead to the degradation of LAMB1, miRanda was used to predict the binding sites between miR-365 and LAMB1 mRNA 3′ UTR (Fig. 6B). To verify the effect of miR-365-1-5p on LAMB1 at the level of translation, the mimic or inhibitor was transfected into MA104 cells. The results showed that the mimic inhibited LAMB1 expression, while the inhibitor increased LAMB1 levels (Fig. 6C). Furthermore, the sequences of the LAMB1 3′ UTR of *Rhesus monkey* containing either the wild-type (WT) or mutant (MUT) binding site for miR-365-1-5p were constructed on the same plasmid. 293T or 293 cells were co-transfected with the corresponding plasmids and the miR-365 mimic/mimic-NC or miR-365-1-5p inhibitor/inhibitor-NC. After 48 h of incubation, firefly and Renilla luciferase activities in these cells were measured. The results showed that miR-365-1-5p could alter the activity of the WT LAMB1 but not that of the MUT LAMB1 3′ UTR (Fig. 6D). Given that miR-365-1-5p enhances RV replication, we hypothesized that LAMB1 may inhibit RV replication. To test this hypothesis, LAMB1-siRNA constructs were generated and transfected into cells, and siRNAs were also used in subsequent experiments (Fig. 6E). Notably, the inhibition of LAMB1 was found to promote RV replication (Fig. 6F).

### DARVR, miR-365-1-5p, and LAMB1 co-regulate C3 during RV infection

**Fig 7:**
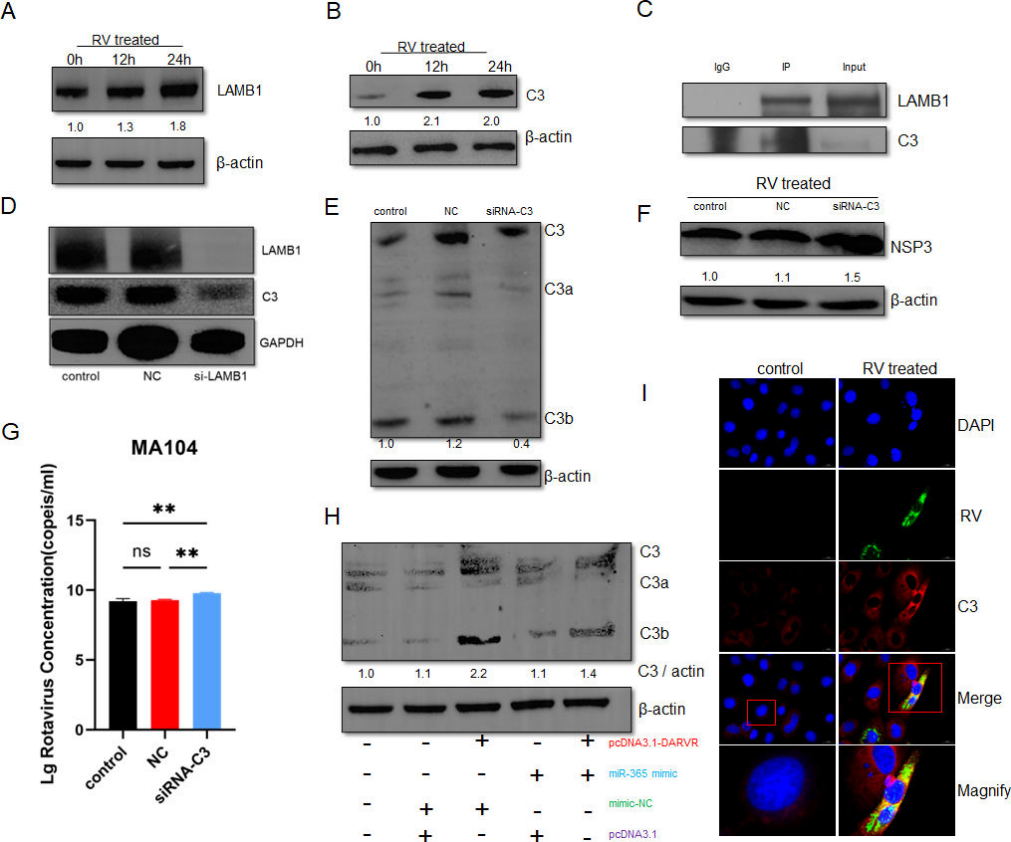
DARVR, miR-365, and LAMB1 co-regulate C3 during RV infection. (A) LAMB1 expression levels at different time points after RV infection were detected by western blotting (ZTR-68, MOI = 0.1). (B) C3 expression levels at different time points after RV infection (ZTR-68, MOI = 0.1). (C) C3 is enriched in the LAMB1 complexes pulled down from the total protein of MA104 cells at 24 h after RV infection, identified using immunoblotting. (D) The effect of LAMB1 inhibition on C3 by RNAi, detected by western blotting. (E) Effect of C3 inhibition was detected by western blotting. (F) Effect of C3 inhibition on RV replication at 16 h post-infection, detected by western blotting (ZTR-68, MOI = 0.1). (G) Effect of LAMB1 inhibition on RV replication at 16 h post-infection, examined using RT-PCR (ZTR-68, MOI = 0.1). (H) Effect of co-transfection or separate transfection of DARVR and miR-365-1-5p mimic on C3, detected by western blotting. (I) Colocalization of RV and C3 (ZTR-68, MOI = 0.1). Comparisons among multiple groups were analyzed via one-way analysis of variance (ANOVA). Data are presented as the mean ± SD of three independent experiments (*P < 0.01).

Studies have shown that LAMB1 plays a role in the complement system and inflammatory responses via interactions with C3. However, there is no evidence that the two interact during RV infection. Thus, we collected whole-cell lysates from MA104 cells infected with RV at 0 h, 12 h, and 24 h post-infection and performed western blot experiments. The results showed that both LAMB1 and C3 were upregulated within 24 h of infection (Fig. 7A and B). To understand whether LAMB1 directly interacted with C3, a co-immunoprecipitation assay was performed. An anti-LAMB1 antibody was used to capture LAMB1 from whole-cell lysates at 24 h post-infection, and an immunoblotting assay for C3 was performed on the pulled-down protein complexes. The results showed that LAMB1 and C3 interact directly with each other (Fig. 7C). Moreover, RNAi against LAMB1 was found to inhibit the expression of C3 (Fig. 7D). To elucidate the regulatory effect of C3 on RV infection, we performed RNAi experiments targeting C3 (Fig. 7E). MA104 cells transfected with RNAi constructs targeting C3 were infected with RV, and whole-cell lysates were obtained at 16 h after RV infection. This lysate was used for immunoblotting and RT-PCR to detect the NSP3 protein of RV. The results showed that NSP3 expression and amount of RV tended to be higher in the RNAi group, demonstrating that C3 inhibition led to increased viral replication (Fig. 7F and G). To verify the effects of DARVR and miR-365-1-5p on C3, DARVR and miR-365-1-5p were co-transfected into MA104 cells. The results showed that miR-365-1-5p inhibited the expression of C3, but DARVR could relieve this inhibition (Fig. 7H). Using multiplex immunofluorescence to label C3 and RV, we revealed the colocalization of RV and C3 during RV infection (Fig. 7I).

### Lnc-DARVR/miR-365-1-5p/LAMB1 axis regulates rotavirus replication via the complement C3 pathway

**Fig 8:**
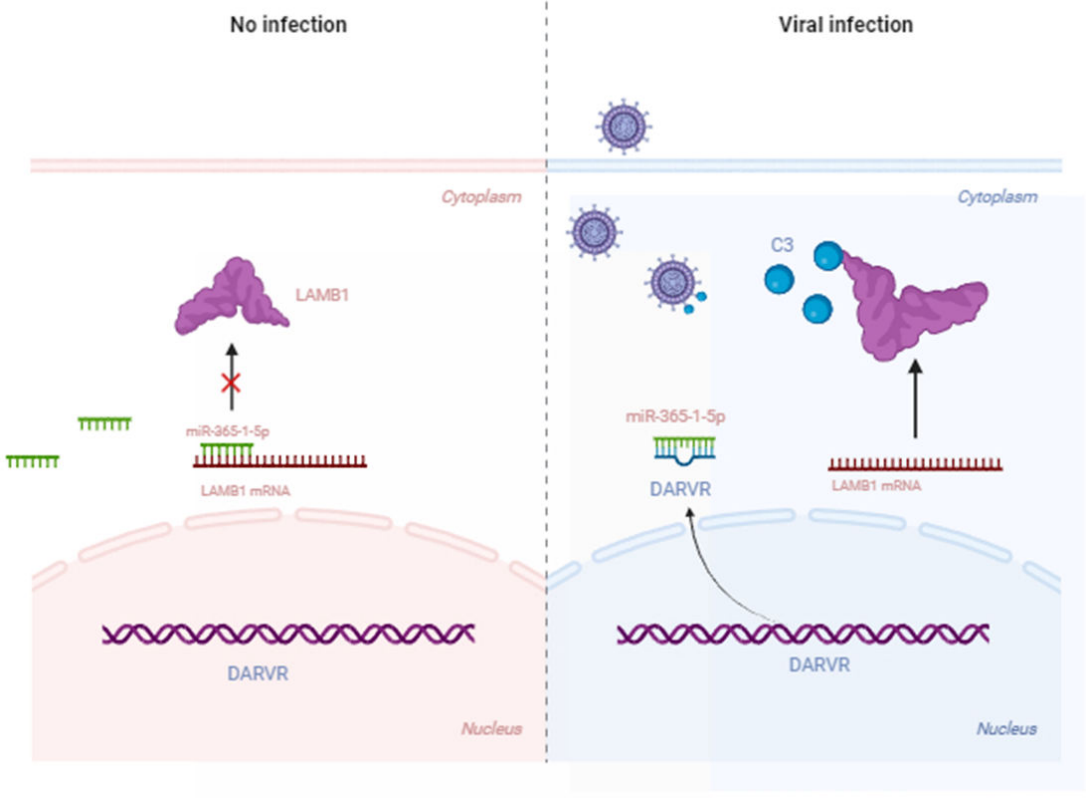
Lnc-DARVR/miR-365-1-5p/LAMB1 axis regulates rotavirus replication via the complement C3 pathway (created with Biorender, https://www.biorender.com/).

DARVR plays an important role in the activation of the complement system during RV infection. miR-365-1-5p inhibits LAMB1, thus suppressing the antiviral response. However, DARVR can regulate the complement system, acting as a ceRNA to sponge off miR-365-1-5p and thereby alleviating its repressive effects on LAMB1 expression, which, in turn, promotes the activity of C3, thereby exerting an antiviral effect (Fig. 8).

## DISCUSSION

Over millions of years of evolution, host cells have developed various antiviral mechanisms, while viruses have concurrently established a wide array of immune evasion strategies. In recent years, lncRNAs have emerged as a focal point of research and have been found to help host cells combat viral infection via host–virus interactions. For example, lnc-ISG20 inhibits the replication of influenza virus by enhancing the expression of ISG-20, although this lncRNA can also be exploited by the virus to promote viral infection (26). VSV promotes its replication by hijacking lncRNA ACOD1 to achieve immune evasion. In this study, we discovered a new lncRNA, which we named DARVR. Our findings showed that this lncRNA plays an antiviral role during RV infection. DARVR is typically located in the nucleus but translocates to the cytoplasm following RV infection. In the cytoplasm, it binds to miR-365-1-5p—a miRNA that can promote RV replication by inhibiting LAMB1 expression. Furthermore, we demonstrated that LAMB1 inhibits RV replication by directly interacting with C3. Thus, our findings suggest that lncRNA DARVR, miR-365-1-5p, and LAMB1 form a regulatory loop through the ceRNA mechanism to jointly regulate the expression of the complement factor C3 (Fig. 8). Nevertheless, how LAMB1 interacts with C3 remains to be explored.

The functions of non-coding RNAs are constantly being uncovered, but the role of miRNAs is fairly well understood. Most miRNAs bind to the 3′ UTR of their target RNAs and promote their degradation, thereby inhibiting their translation (24). In this study, dual luciferase reporter gene experiments demonstrated that miR-365 had little effect on LAMB1 mRNA 3′ UTR, which may be because miR-365 may also affect the expression of LAMB1 protein through other pathways. Studies have shown that RV infection alters the miRNA expression profile of host cells. Bovine RV can alter miR-365 expression in MA104 cells, and HSBP6 is co-expressed with miR-365 (25). Specifically, miR-4301 promotes RV replication through PPP1R3D, while miRNA-7 inhibits RV replication by targeting the RV NSP5 protein (27, 28). Notably, miRNAs often have multiple target genes. Gene interactions constitute a complex regulatory network that does not function in isolation. Herein, we found that miR-365-1-5p has multiple targets. In this article, miR-365-1-5p mimics promoted rotavirus replication, while its inhibitor inhibited rotavirus replication, with a smaller effect. The reason may be that miR-365-1-5p needs to be infected with rotavirus at a limited MOI to produce more obvious differences compared with the control group. Future studies on low MOI rotavirus infection will help to gain a deeper understanding of this effect. Although the RNA interference experiment of LAMB1 seemed to have little effect on the replication of the rotavirus genome, this may be because the infection MOI of rotavirus was too high, and rotavirus infection with a lower MOI may make this experiment biologically meaningful. Importantly, it interacts with lnc-DARVR, thereby affecting the expression of LAMB1 in the host and modulating the antiviral response. DARVR acts as a molecular sponge and sequesters miR-365-1-5p, thereby maintaining LAMB1 expression, which, in turn, promotes the activity of complement factor C3 and enables the inhibition of RV replication. Collectively, these results indicate that DARVR achieves antiviral effects by inhibiting the regulation of the target genes of miR-365-1-5p. This reveals the presence of a regulatory loop through which this lncRNA protects against viral infection by promoting complement C3 activity.

Laminins α, β, and γ collectively constitute laminin, with each laminin chain representing a multidomain protein encoded by a distinct gene. However, the specific functions of different chains and the different chain-forming trimeric molecules remain largely unclear. Most of the current research on LAMB1 focuses on its role in cancer (18). Some studies have shown that LAMB1 may be involved in inflammation. Excessive hydrogen sulfide exposure promotes C9 activity and inhibits LAMB1, thereby causing brain damage (29). This suggests that LAMB1 may interact with the complement system. In this study, we demonstrated the interaction between LAMB1 and the complement factor C3 after RV infection, and we found that LAMB1 can promote the expression of C3. Although the RNA interference experiment of LAMB1 seemed to have little effect on the replication of the rotavirus genome, this may be because the infection MOI of rotavirus was too high, and rotavirus infection with a lower MOI may make this experiment biologically meaningful. However, whether C3 retains its activity in the absence of LAMB1 remains unclear, and how LAMB1 promotes the expression or function of C3 warrants further investigation.

The complement system can block viral infection, and complement C4 inhibits adenovirus infection by inactivating the viral capsid (30). However, viruses can encode complement regulators to achieve immune escape (31). The three pathways required for activating the complement system have been delineated, but the protein interactions of the complement system are still being explored. We discovered that LAMB1 can interact with C3, and RNAi against LAMB1 can attenuate the antiviral effect of C3. Studies have shown that C3 and LAMB1 jointly contribute to host inflammatory response. However, research on the function of C3 and LAMB1 in viral infection is fairly limited. Nevertheless, our findings revealed that there exists a direct interaction between LAMB1 and C3, although the mechanism of this interaction is unclear. LAMB1 is a target gene of miR-365-1-5p, and therefore, it should be downregulated when miR-365-1-5p is upregulated during RV infection. However, the nuclear export of DARVR enables the sequestration of miR-365-1-5p, preventing the downregulation of LAMB1 via the ceRNA mechanism.

Anyway, the interaction between C3 and LAMB1 remains unknown, and we only provide evidence for their direct interaction. The mechanism by which C3 inhibits rotavirus replication also remains unclear. Future studies on DARVR-deficient cells may provide more insights into the interaction between C3 and rotavirus. Further investigation is needed to uncover these mechanisms and interactions. Rotavirus is a double-stranded RNA virus, and it remains to be explored whether the regulatory mechanism mediated by lnc-DARVR, miR-365-1-5p, and LAMB1 will respond to other single-stranded RNA viruses and DNA viruses.

In summary, our study revealed that LAMB1 plays an important role in the activation of the complement system during RV infection. miR-365-1-5p inhibits LAMB1, thus suppressing the antiviral response. However, DARVR can regulate the complement system, acting as a ceRNA to sponge off miR-365-1-5p, thereby alleviating its repressive effects on LAMB1 expression. Thus, it inhibits RV replication during the early stage of infection. Our findings highlight the critical role of lncRNAs in virus–host interactions and illustrate a ceRNA-based regulatory mechanism within the complement system.

### Conclusions

In summary, we identified a previously unannotated lncRNA and named it DARVR. During rotavirus infection, DARVR adsorbs miR-365-1-5p, alleviating its inhibitory effect on LAMB1. Furthermore, complement factor C3 requires LAMB1 to exert its anti-rotavirus replication effects. Notably, our study enhances the understanding of the antiviral functions of lncRNAs by demonstrating their role in promoting host cytokines, thereby offering new directions for the design of antiviral drugs.

## Data Availability

Data are contained within the article.
